# Evaluation of Efficacy of Neoadjuvant Immunochemotherapy for Cervical Metastatic Squamous Cell Carcinoma of Unknown Primary

**DOI:** 10.1002/cam4.70869

**Published:** 2025-05-01

**Authors:** Xianlu Gao, Shuwen Fu, Chulin Yang, Wenmei Jiang, Jiadong Liang, Ruiyu Li, Shida Yan, Mingyuan Du, Shiyan Yang, Quan Zhang, Shuwei Chen

**Affiliations:** ^1^ Department of Head and Neck Surgery Sun Yat‐Sen University Cancer Center Guangzhou China; ^2^ State Key Laboratory of Oncology in South China Guangzhou China; ^3^ Guangdong Provincial Clinical Research Center for Cancer Guangzhou China; ^4^ Department of Anesthesiology Hospital of Stomatology, Sun Yat‐Sen University Guangzhou China; ^5^ Guanghua School of Stomatology, Sun Yat‐Sen University Guangzhou China; ^6^ Department of Thyroid Surgery, Zhejiang Cancer Hospital, Hangzhou Institute of Medicine Chinese Academy of Sciences Hangzhou China

**Keywords:** cervical metastatic squamous cell carcinoma of unknown primary, immunotherapy, neoadjuvant therapy, PD‐1 inhibitors, squamous cell carcinoma of the head and neck

## Abstract

**Purpose:**

This study aims to evaluate the antitumor activity of neoadjuvant immunochemotherapy in patients with cervical metastatic squamous cell carcinoma of unknown primary (CMSCCUP), providing a new idea for its treatment.

**Method:**

We retrospectively examined the medical records from Sun Yat‐sen University Cancer Center to identify patients with CMSCCUP from July 2020 to November 2023. CMSCCUP's diagnoses are based on the results of biopsy and imaging examination. All patients received PD‐1 inhibitors combined with chemotherapy: paclitaxel (Albumin‐bound) 260 mg/m^2^ and cisplatin 60 mg/m^2^ every 3 weeks for two or more cycles as their initial treatment. Clinical response after neoadjuvant immunochemotherapy was assessed using Response Evaluation Criteria in Solid Tumors (RECIST), version 1.1. Surgery, radiotherapy, or chemoradiotherapy was performed subsequently based on the imaging evaluation results. Outcomes included overall survival (OS) and disease‐free survival (DFS).

**Result:**

After neoadjuvant immunochemotherapy, the objective response rate (ORR) was 84.2%; complete response (CR), partial response (PR), and stable disease (SD) were 36.8%, 47.4%, and 15.8%, respectively. Of the 9 patients (42.9%) who underwent surgery after neoadjuvant immunochemotherapy, 7 (77.8%) achieved pathological complete response (pCR). No severe treatment‐related adverse events occurred. The most common treatment‐related adverse events were Grade 1–2 fatigue (11/14, 78.6%), decreased appetite (11/15, 73.3%), and increased aspartate transaminase (12/20, 60.0%). One (1/20, 5.0%) patient experienced Grade 3 anemia. The 2‐year overall survival (OS) and 2‐year disease‐free survival (DFS) rates were 94.4% and 89.4%, respectively.

**Conclusion:**

Neoadjuvant immunochemotherapy showed favorable efficacies, anticipated outcomes, and tolerable toxicity for patients with CMSCCUP. More clinical practice and clinical studies are still needed to confirm further.

## 1 Introduction

Cervical metastatic squamous cell carcinoma of unknown primary (CMSCCUP) is defined as the histological diagnosis of squamous cell carcinoma metastasis while the primary lesion fails to be detected after detailed physical examination, endoscopy, or imaging, accounting for about 2%–5% of all head and neck carcinoma [1]. Due to the slow progression, small size, and the hidden location of the primary tumor (mostly located in the Waldeyer's ring and the base of tongue) [2, 3], it is difficult to diagnose, slowing down the development of treatments for CMSCCUP. Several recommendations aiming at treating CMSCCUP are provided, including surgery, radiotherapy, chemotherapy, and surgery ± radiotherapy or chemotherapy [4]. Despite advances in the treatment of CMSCCUP, survival rates with currently available therapies in CMSCCUP are dismal, with a 5‐year survival rate of less than 40% [4, 5, 6, 7].

Immune checkpoint plays a key role in the tumor microenvironment and can serve as a mechanism of immune escape, leading to the development and occurrence of cancers [8, 9]. In recent years, clinicians' understanding of biological mechanisms of immune escape and implications of immunotherapy in head and neck squamous cell carcinoma (HNSCC) has gradually deepened. PD‐1 inhibitors, such as pembrolizumab, nivolumab, and atezolizumab, have shown efficacy and manageable safety in recurrent or metastatic HNSCC [10, 11, 12]. Neoadjuvant immunotherapy with PD‐1 inhibitors before surgery can reduce the size of the lesion, facilitate surgery, decrease the likelihood of postoperative complications, and improve the quality of life of patients. While neoadjuvant immunotherapy for CMSCCUP is still rarely performed and controversial, several institutions have conducted clinical studies and confirmed that PD‐1 inhibitors had encouraging efficacy as well as an acceptable safety profile [13, 14]. The NCCN [5] guidelines recommend neck dissection as a standard treatment option for patients with CMSCCUP. For patients with a pathological stage of pN1 without extranodal extension (ENE), postoperative radiotherapy or close observation is recommended; for those with pN2–pN3 without ENE, postoperative radiotherapy or systemic therapy is advised. In cases with ENE, radiation + systemic therapy or concurrent chemoradiation should be considered. Cisplatin is recommended as the preferred regimen for postoperative systemic therapy. In patients who are ineligible for cisplatin or present with positive margins and/or ENE, a regimen of docetaxel combined with cetuximab (Category 2B) may be considered. However, immunochemotherapy has achieved good efficacy in recurrent and locally advanced head and neck squamous cell carcinoma, which can reduce the size of the primary lesion and lymph nodes, facilitate the operation, and lead to a good prognosis [11, 12]. Besides, several phase II clinical studies have evaluated the combination of neoadjuvant immunotherapy and chemotherapy in resectable head and neck squamous cell carcinoma, yielding objective outcomes. NCT02296684 [15], NCT02641093 [16], and NCT04826679 [17], and other Phase II clinical studies have confirmed the efficacy and safety of neoadjuvant immunotherapy in resectable locally advanced head and neck squamous cell carcinoma, demonstrating objective pathologic responses. Moreover, the unknown primary tumor location poses significant challenges in treatment planning. Unlike cases where the primary site is identified and can be directly resected, CMSCCUP lacks a clearly defined tumor origin, making direct mucosal resection “blind,” which potentially leads to residual tumor and significantly increases the risk of postoperative recurrence. Additionally, the mucosal surfaces of the head and neck, including the nasopharynx, oropharynx, and hypopharynx, are extensive, and widespread resection could lead to irreversible functional impairments such as swallowing and speech difficulties. Furthermore, CMSCCUP often exhibits an insidious disease course with potential micrometastases or systemic dissemination at diagnosis. Local treatment alone (surgery or radiotherapy) may be insufficient for disease control, necessitating systemic therapy. Here, we aimed to determine whether PD‐1 inhibitors improved overall survival (OS) and evaluate their efficacy and safety in participation with CMSCCUP.

## 2 Methods

### 2.1 Participations

**FIGURE 1:**
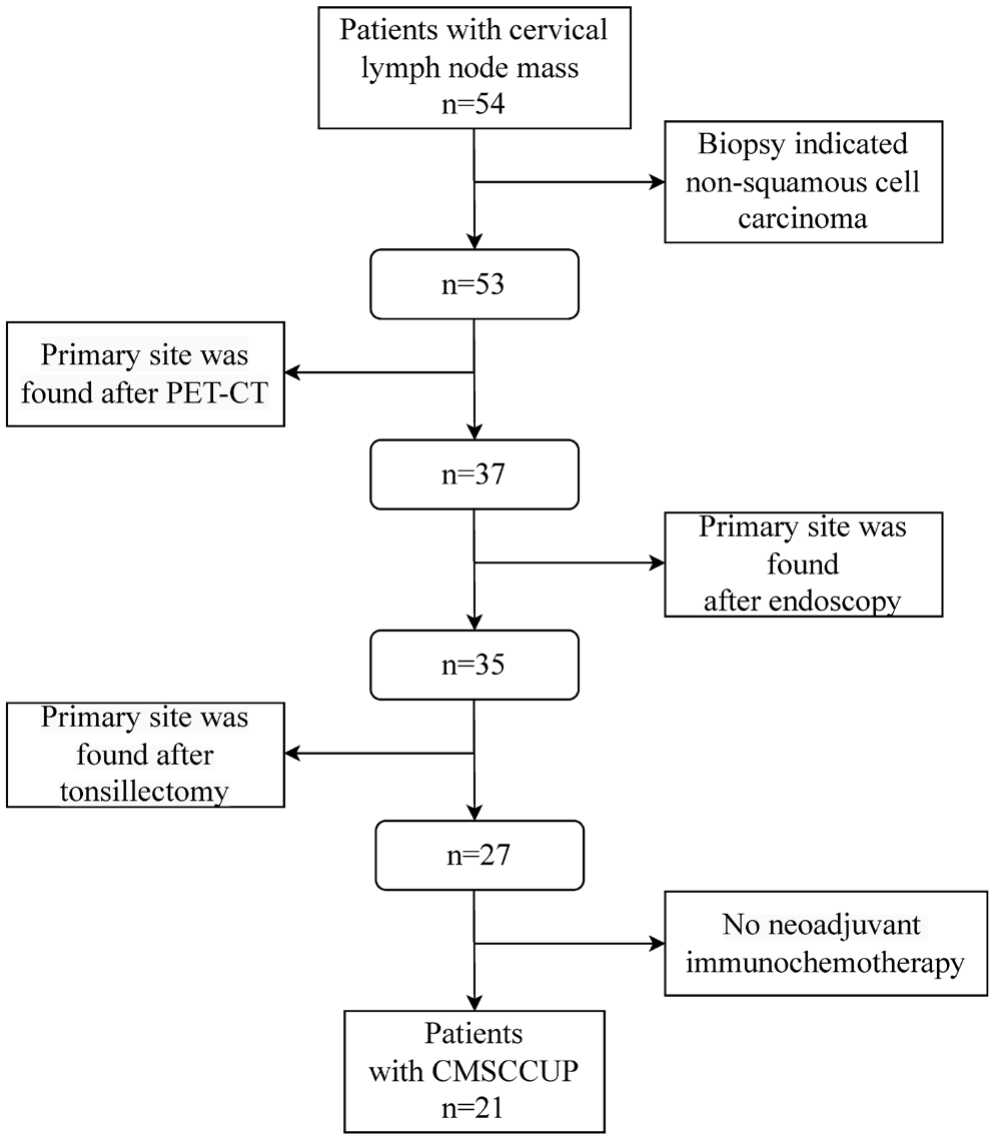
Flow chart of patient screening: A total of 54 patients presenting with cervical lymph node metastasis were initially evaluated. After biopsy, detailed imaging, endoscopy, tonsillectomy, and exclusion of non‐neoadjuvant immunochemotherapy, 21 CMSCCUP patients were finally enrolled.

We retrospectively examined the medical records from Sun Yat‐sen University Cancer Center to identify patients with CMSCCUP from July 2020 to November 2023. Twenty‐one patients who had biopsy‐confirmed squamous cell carcinoma (SCC) limited to the cervical lymph nodes without an identifiable primary site and received neoadjuvant immunochemotherapy at Sun Yat‐Sen University Cancer Center were eligible for the present study. CMSCCUP diagnostics were based on the results of either fine‐needle aspiration or excisional biopsy. Pretreatment initial diagnostic workup included detailed physical examination, endoscopic examination, positron emission tomography with computed tomography (PET‐CT), contrast‐enhanced CT, and/or magnetic resonance imaging (MRI). Patients were included if they met the following criteria: histologically confirmed cervical lymph node squamous cell carcinoma (SCC) based on biopsy, age between 18 and 80 years old, no identified primary tumor after pretreatment initial diagnostic workup, received neoadjuvant immunochemotherapy at our institution and had no distant metastasis. Exclusion criteria included non‐SCC histology, age < 18 or > 80 years, detection of a primary tumor upon examination, failure to receive neoadjuvant immunochemotherapy, or evidence of distant metastasis. The clinical stage was assigned retrospectively according to the American Joint Committee on Cancer (AJCC) Staging Manual, 8th edition [18]. The flow chart of patient screening is shown in Figure 1.

### 2.2 Treatments

After diagnosis and staging, all patients received PD‐1 inhibitors combined with chemotherapy: paclitaxel (albumin bound) 260 mg/m^2^ and cisplatin 60 mg/m^2^ every 3 weeks for two or more cycles as their initial treatment. Imaging examinations were performed after or during the initial treatment, and response to treatment was assessed using RECIST, version 1.1 [19]. The primary endpoint of the study was objective response rate (ORR), which was defined as the proportion of patients with a confirmed complete response (CR) or partial response (PR). For patients who achieve radiographic complete response (CR) or partial response (PR) after neoadjuvant immunochemotherapy, surgery is generally the preferred treatment option. Surgery not only allows for the complete removal of residual tumors and reduces the risk of recurrence due to ENE but also provides a pathological assessment of tumor response. If a pathological complete response (pCR) is achieved, postoperative adjuvant therapy may be omitted, thereby minimizing treatment‐related toxicity. Active surveillance will be implemented to identify occult primary lesions and to promptly address disease recurrence or metastatic progression. Conversely, if significant residual tumor remains or high‐risk features are present—such as ENE, multiple lymph node metastases, moderately/poorly differentiated, or HPV negative status—adjuvant radiotherapy may be necessary to enhance disease control. For cases where the tumor invades critical structures (e.g., carotid artery, cranial nerves, and prevertebral space) or exhibits extensive soft tissue infiltration, making an R0 resection unfeasible, radiotherapy is the preferred approach. Ultimately, the choice between surgery and radiotherapy should also consider patient preferences and financial factors to develop an individualized treatment plan.

### 2.3 Response Evaluation

Hematoxylin–eosin‐stained (H&E‐stained) and formalin‐fixed paraffin‐embedded (FFPE) sections of surgical specimens were applied for assessing pathologic responses by two experienced pathologists. The US Food and Drug Administration (FDA) issued the position statement: ‘Pathologic complete response (pCR) is defined as the absence of residual invasive cancer… after evaluation of the completely resected specimen including …all sampled regional lymph nodes following completion of neoadjuvant systemic therapy’. The FDA additionally designated pCR as a surrogate endpoint [20]. Treatment‐related adverse events are graded according to the National Cancer Institute Common Terminology Criteria for Adverse Events, version 4.0 [21].

Patients were identified according to their electronic medical records. This study was approved by the ethics committee of Sun Yat‐sen University Cancer Center, and written informed consent was provided by all the patients. This study is in line with the Strengthening the Reporting of Observational Studies in Epidemiology (STROBE) guidelines.

### 2.4 Statistical Analysis

All statistical analyses were calculated using R, version 4.3.1. Overall survival (OS) was defined as the time from the first time of initial treatment until the date of death from any cause. Disease‐free survival (DFS) was defined as the time from the first time of initial treatment until the date of radiographic disease progression or death. OS and DFS were calculated using Kaplan–Meier methods. Moreover, multivariate and univariate Cox regression were used to examine the refined relationships between essential variables. These factors included age, sex, smoking history, alcohol history, tonsillectomy, HPV status, differentiation, diameter of the largest node, and TNM staging. A *p* value threshold of 0.05 was used for statistical significance.

## 3 Result

### 3.1 Patient Characteristics

From 2020 July to 2023 November, 21 patients were diagnosed with CMSCCUP; patients and tumor characteristics of the patients are presented in Table 1. The median age of the cohort (*n* = 21) was 58 years (range 29–79 years), and the majority of the patients were male (90.5%, 19/21). All patients received detailed physical examinations, among which 17 (81.0%) received endoscopy. All patients underwent comprehensive imaging examinations, including one, two, or all three modalities: CT, MRI, and PET‐CT. According to imaging reports, the most common site of nodal involvement was Level II (85.7%), followed by Level III (76.2%) and Level IV (57.1%). For those who had radiographic suspicion of tonsil abnormality, unilateral tonsillectomy was performed in three patients and bilateral tonsillectomy in one patient. However, the final pathological findings did not reveal cancer. Among the patients, only four were HPV positive; yet in 47.6% (10/21), the testing was not performed. Patients were staged according to the 8th edition of AJCC staging criteria; most patients were classified as cN2 (61.9%, 13/21). According to fine needle aspiration biopsy before treatment, all were squamous cell carcinoma, and the majority (13/21, 72.2%) were poorly differentiated.

**TABLE 1:** Baseline characteristics (*N* = 21).

Variables		*N* (%)
Age, median (range)		58 (29–79)
Gender (%)	Male	19 (90.5)
	Female	2 (9.5)
Smoking (%)	No	9 (42.9)
	Yes	12 (57.1)
Alcohol (%)	No	11 (52.4)
	Yes	10 (47.6)
Physical examination (%)	Yes	21 (100.0)
Endoscopy (%)	No	4 (19.0)
	Yes	17 (81.0)
Ultrasound (%)	No	7 (33.3)
	Yes	14 (66.7)
CT (%)	No	14 (66.7)
	Yes	7 (33.3)
MR (%)	No	10 (47.6)
	Yes	11 (52.4)
PET (%)	No	5 (23.8)
	Yes	16 (76.2)
Biopsy (%)	Yes	21 (100.0)
Tonsillectomy (%)	No	17 (81.0)
	Unilateral	3 (14.3)
	Bilateral	1 (4.8)
p16 (%)	No	12 (57.1)
	Positive	4 (19.0)
	Negative	5 (23.8)
HPV (%)	No	17 (81.0)
	HPV16	1 (4.8)
	Negative	3 (14.3)
PD1 detection (%)	No	19 (90.5)
	Yes	2 (9.5)
cN (%)	cN1	2 (9.5)
	cN2b	11 (52.4)
	cN2c	2 (9.5)
	cN3a	1 (4.8)
	cN3b	5 (23.8)
Stage (%)	III	2 (9.5)
	IVa	13 (61.9)
	IVb	6 (28.6)
Malignant lymph nodes short‐axis diameter (mean (SD))		30.55 (11.27)
Differentiation (%)	Poorly	13 (72.2)
	Moderately	4 (22.2)
	Highly	1 (5.6)

### 3.2 Treatments and Outcomes

Treatment‐related characteristics are presented in Table 2. All the patients (*n* = 21) were treated with PD‐1 inhibitors combined with albumin‐bound paclitaxel and cisplatin (TP regimen). Most patients (20/21, 95.2%) received two or more courses of neoadjuvant immunochemotherapy while one patient was treated only once owing to a force majeure factor. Most patients underwent imaging examination after or during neoadjuvant immunochemotherapy to assess the next treatment. Due to the lack of authoritative treatment for CMSCCUP, clinicians proceed with the next treatment based on the multiple disciplinary team (MDT), clinical experience, and the patient's willingness. After neoadjuvant immunochemotherapy, five (23.8%) patients underwent cervical lymph node dissection; one of them received an immunotherapy session after surgery, eight (38.1%) patients underwent radiotherapy/chemoradiotherapy, four (19.0%) patients underwent cervical lymph node dissection combined with radiotherapy/chemoradiotherapy, and two of them were also treated with immunotherapy. One patient (4.8%) underwent immune maintenance therapy instead of surgery or chemoradiotherapy, and the remaining three (14.3%) patients were followed up closely after neoadjuvant immunochemotherapy without subsequent treatments. Of the nine patients (42.9%) who underwent surgery after neoadjuvant immunochemotherapy, seven (77.8%) achieved pathological complete response (pCR). Treatment paths are shown in Table 3.

**TABLE 2:** Treatment‐related characteristics (*N*=21).

Variables		*N*
Immunotherapy regimen (%)	Pembrolizumab	6 (28.6)
	Nivolumab	2 (9.5)
	Tislelizumab	4 (19.0)
	Toripalimab	1 (4.8)
	Sintilimab	2 (9.5)
	Camrelizumab	6 (28.6)
Neoadjuvant immunochemotherapy cycle (%)	1	1 (4.8)
	2	6 (28.6)
	3	9 (42.9)
	4	5 (23.8)
Surgery (%)	No	12 (57.1)
	Yes	9 (42.9)
Delay of surgery (mean (range))		24.6 (17.0–36.0)
Adjuvant radiotherapy (%)	No	9 (42.9)
	Yes	12 (57.1)
RECIST (%)	CR	7 (36.8)
	PR	9 (47.4)
	SD	3 (15.8)

**TABLE 3:** Treatment paths of each patient.

ID	Gender	Age	HPV status	cN	cTNM stage	Neoadjuvant immunochemotherapy cycle	RECIST	Surgery	pN	pTNM stage	Differentiation	Adjuvant chemoradiotherapy	Adjuvant immunotherapy	Outcome
1	Male	64	NA	cN2b	IVa	2	CR	Yes	pN0	pCR	Poorly	Yes	No	Alive
2	Male	51	Negative	cN2b	IVa	4	PR	Yes	pN0	pCR	Moderately	Yes	Yes	Alive
3	Male	51	NA	cN2b	IVa	3	CR	Yes	pN0	pCR	NA	Yes	Yes	Alive
4	Male	65	NA	cN2b	IVa	3	CR	No	—	—	Poorly	No	Yes	Alive
5	Male	34	Negative	cN3b	IVb	1	PR	Yes	pN3b	IVb	Highly	Yes	No	Recurrence
6	Male	57	NA	cN3b	IVb	3	NA	No	—	—	Poorly	No	No	Death
7	Male	52	Negative	cN2b	IVa	4	SD	No	—	—	Poorly	Yes	No	Alive
8	Male	48	Positive	cN2b	IVa	4	PR	No	—	—	NA	Yes	No	Alive
9	Male	79	Negative	cN3b	IVb	2	PR	No	—	—	Poorly	Yes	No	Alive
10	Male	62	Positive	cN2b	IVa	2	PR	Yes	pN0	pCR	Poorly	No	Yes	Alive
11	Male	53	Negative	cN2b	IVa	4	PR	No	—	—	Moderately	Yes	No	Alive
12	Male	64	NA	cN3b	IVb	3	PR	Yes	pN0	pCR	Poorly	No	No	Alive
13	Male	58	Negative	cN2b	IVa	3	CR	No	—	—	Poorly	Yes	No	Alive
14	Male	59	NA	cN2c	IVa	3	SD	No	—	—	Poorly	No	No	Alive
15	Male	73	Positive	cN2b	IVa	2	CR	Yes	pN0	pCR	Poorly	No	No	Alive
16	Male	57	Negative	cN2c	IVa	3	CR	No	—	—	Poorly	Yes	No	Alive
17	Male	68	NA	cN1	III	2	NA	No	—	—	Moderately	No	No	Alive
18	Female	29	NA	cN2b	IVa	3	PR	Yes	pN1	III	NA	No	No	Alive
19	Male	74	NA	cN3a	IVb	3	SD	No	—	—	Moderately	Yes	No	Alive
20	Female	49	Positive	cN1	III	2	CR	Yes	pN0	pCR	Poorly	No	No	Alive
21	Male	71	NA	cN3b	IVb	4	PR	No	—	—	Poorly	Yes	No	Alive

**FIGURE 2:**
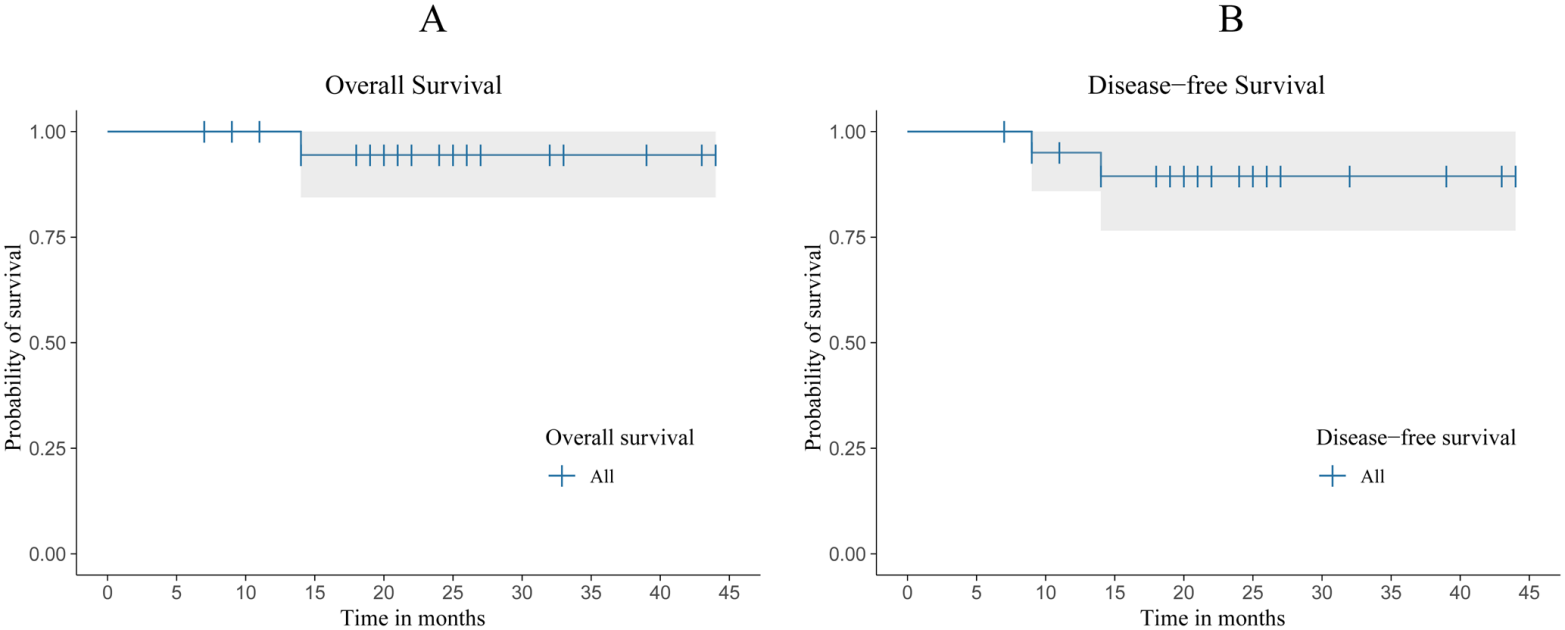
Kaplan–Meier survival analysis of the study. 2A, Overall survival (OS) curve of the cohort, The 2‐year OS rate of the cohort was 94.4%. 2B, Disease‐free survival (DFS) curve of the cohort. The 2‐year DFS rate of the cohort was 89.4%.

**FIGURE 3:**
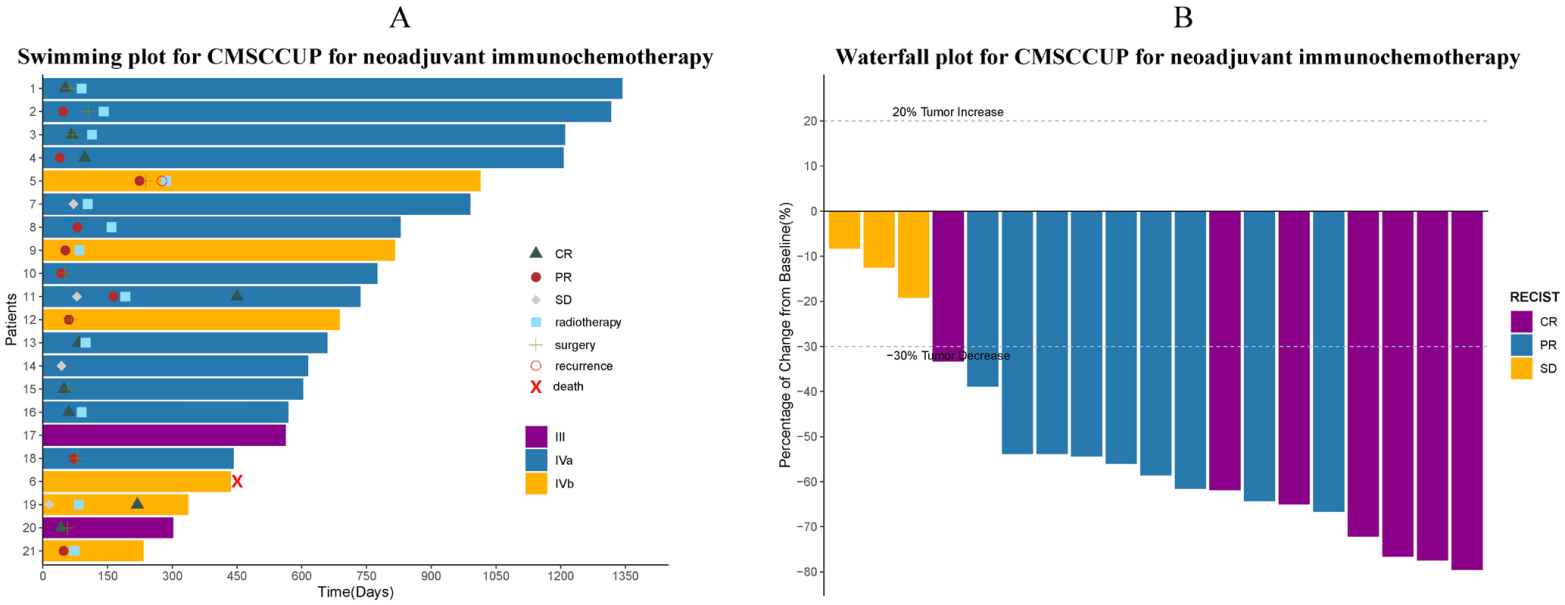
3A, Swimming plot illustrating posttherapy response and response duration. 3B, Waterfall plot illustrating radiographic response after neoadjuvant immunochemotherapy based on RECIST v1.1 criteria. CR, complete response; PR, partial response; SD, stable disease.

At data cut‐off on May 13th, 2024, the median follow‐up for the evaluable cohort (*N* = 21) was 22 months (7–44 months). Data show a 2‐year OS rate of 94.4% and a 2‐year DFS rate of 89.4% (Figure 2). After receiving neoadjuvant immunochemotherapy, 19 patients' imaging results before and after treatment were compared, showing stable disease (SD) in three (15.8%) patients, partial response (PR) in nine (47.4%), and complete response (CR) in seven (36.8%), yielding an ORR of 84.2% (Figure 3). Univariate or multivariate Cox regression showed no correlation with prognosis.

### 3.3 Safety

The most common treatment‐related adverse events were Grade 1–2 fatigue (11/14, 78.6%), decreased appetite (11/15, 73.3%), and increased aspartate transaminase (12/20, 60.0%). One (1/20, 5.0%) patient experienced Grade 3 anemia. Follow‐up time ranged from 7 to 44 months (median follow‐up time was 22 months). The 2‐year overall survival (OS) and 2‐year disease‐free survival (DFS) rates were 94.4% and 89.4%, respectively.

## 4 Discussion

This is a retrospective study of the treatment of CMSCCUP, which has rarely been mentioned before, focusing on the efficacy of neoadjuvant immunochemotherapy. To date, the treatment of CMSCCUP is currently lacking authoritative standards and is still controversial. Several institutions have recommended aggressive therapeutic protocols for CMSCCUP. The general characteristics of our patients are similar to those in other literature: most of the patients were male (90.5%), 61.9% of the clinical lymph nodes were N2, and the affected lymph nodes were Level II (85.7%) and Level III (76.2%). To confirm CMSCCUP, a series of examinations were performed on the patient, and the diagnosis was made when the FNA indicated cervical lymph node metastasis and no primary site was found on any imaging. The study by Rusthoven et al. [22] stated that in approximately 25% of cases, FDG‐PET detected primary tumors that could not be detected otherwise, and FDG‐PET was sensitive to previously unrecognized regional or distant metastases in 27% of cases. In our study, 72.7% of patients underwent FDG‐PET, which also enhanced the diagnosis of CMSCCUP.

The purpose of this study is to explore an alternative treatment for CMSCCUP. Miller's study found that of the seven patients with pN1 who received selective neck dissection (SND) alone, six did not relapse at a mean follow‐up of 41.2 months after SND, while only one of the patients with ENE relapsed at 11 months after surgery [23]. Most of the studies suggest that surgery alone is sufficient, unless mucosal lesions, which can be supplemented with mucosal irradiation or unilateral neck irradiation. According to recent study, 350 CMSCCUP patients were treated with either unilateral or bilateral radiation therapy, showing that 3‐year local, regional, locoregional failure rates and CUP‐specific survival were 5.6%, 11.7%, 15.0%, and 84.7%, respectively [24]. Another study showed the 5‐year DFS and OS of 60% and 51.2%, respectively, in the total 80 patient population [25]. In addition, Beldì's study [26] showed a 5‐year OS of 46.6% for the subgroup with squamous cell carcinoma histology after radiotherapy. Compared to the above radiotherapy, our study demonstrated an ORR, 2‐year OS, and 2‐year DFS of 84.2%, 94.4%, and 89.4%, respectively, showing a better therapeutic effect and prognosis.

In recent years, immunotherapy has been applied in recurrent and metastatic head and neck squamous cell carcinoma, and some scholars have carried out clinical studies to explore the application of immunotherapy in CMSCCUP. J. Tanizak [14] launched an open‐label Phase II study, which showed an ORR, median PFS, and OS of 22.2% (95% CI, 11.2%–37.1%), 4.0 months (95% CI, 1.9–5.8 months) and 15.9 months (95% CI, 8.4–21.5 months), respectively, for the 45 previously treated patients. Similar clinical benefits were observed in 11 previously untreated patients. Another clinical study initiated by Raghav showed similar results [13]. Compared with previous studies, our study shows favorable outcomes. Notably, of the patients (9/21, 42.9%) who underwent surgery after neoadjuvant immunochemotherapy, seven achieved pCR.

Treatment‐related adverse events are also noteworthy. PD‐1 inhibitor was safe and well tolerated. Refer to previous studies; anemia and nausea were the most common adverse events of immunotherapy. KEYNOTE‐012 [11], an open‐label, multicenter, Phase Ib trial provided compelling evidence: treatment‐related adverse events occurred in 63% of patients, the most common being Grade 1–2 pruritus, fatigue, or transient rash. Grade 3–5 treatment‐related adverse events occurred in 17% of patients. In our cohort, the most common treatment‐related adverse events were Grade 1–2 fatigue, decreased appetite, and AST increase; only one patient had Grade 3 anemia. No severe toxic effects were noted.

Human papillomavirus (HPV), specifically Type 16, is associated with the development of a subgroup of squamous cell carcinomas of the head and neck; almost 25.9% of HNSCC have been attributed to HPV infection [27]. For CMSCCUP, HPV detection not only suggests a better prognosis but also could be used as a surrogate marker in the search for the primary site [28]. Our institution used real‐time polymerase chain reaction (PCR) to measure HPV viral load and/or immunohistochemistry to detect p16 expression. For HPV detection in 11 (52.4%) patients, 4 (36.4%) patients were HPV‐associated CMSCCUP. However, no correlation between HPV status and OS or DFS was found in univariate regression analysis. More cases are needed to validate the correlation.

Our study has certain limitations due to its small sample size and single‐center design, which lends itself to referral and selection biases that can mar generalizability to an unselected patient population with CMSCCUP. Larger studies are required to further these findings. However, the single‐center enrollment did allow us to ensure the fidelity of CMSCCUP diagnosis with rigorous review. There are questions remaining to be solved: when single‐modality therapy is sufficient; whether to de‐escalate treatment for HPV‐positive patients; and whether to undergo surgery or radiotherapy after immunotherapy. HPV status may influence treatment decisions and outcomes. Further studies are needed to determine optimal treatment strategies based on HPV status and post‐treatment responses.

In conclusion, neoadjuvant immunochemotherapy provides a well‐tolerated treatment with encouraging (although somewhat limited) efficacy. More multicenter randomized controlled trials with larger cohorts are urgently needed, which will help confirm our findings and determine the true therapeutic potential of this regimen in CMSCCUP.

## Author Contributions

Xianlu Gao contributed to the conceptualization, methodology, software development, data curation, investigation, visualization, and writing of the original draft. Shuwen Fu contributed to methodology, data curation, and investigation. Chulin Yang contributed to methodology, data curation, and investigation. Wenmei Jiang, Jiadong Liang, and Ruiyu Li contributed to validation, formal analysis, and writing – review and editing. Shiyan Yang contributed to methodology, data curation, and investigation. Shida Yan contributed to validation, formal analysis, and writing – review and editing. Mingyuan Du contributed to validation, funding acquisition, and writing – review and editing. Quan Zhang contributed to supervision, funding acquisition, project administration, resources, and writing – review and editing. Shuwei Chen contributed to conceptualization, supervision, funding acquisition, project administration, resources, and writing – review and editing.

## Ethics Statement

The study protocol was approved by the Institutional Research Ethics Committee of Sun Yat‐sen University Cancer Center (SL‐B2024‐605‐01).

## Consent

The authors have nothing to report.

## Conflicts of Interest

The authors declare no conflicts of interest.

## Data Availability

The data that support the findings of this study are available from the corresponding author upon reasonable request.
